# Case study: Body composition and bone changes following a thru‐hike of the Pacific Crest Trail by a young female adult

**DOI:** 10.14814/phy2.71058

**Published:** 2026-08-09

**Authors:** Dale R. Wagner, Edward M. Heath, Macy Gustavus

**Affiliations:** ^1^ Kinesiology and Health Science Utah State University Logan Utah USA; ^2^ Campus Recreation Utah State University Logan Utah USA

**Keywords:** backpacking, bone density, dual‐energy x‐ray absorptiometry, hiking, RED‐S, visceral adipose tissue

## Abstract

A 24‐year‐old premenopausal female adult completed the entire Pacific Crest Trail, an arduous backpacking thru‐hike of 4265 km. Dual‐energy x‐ray absorptiometry measurements were taken before and after the trek. Her body fat percentage dropped from 33.2% to 24.9%, and there was a 52% decrease in visceral adipose tissue mass and area. Total body bone mineral content and bone mineral density (BMD) were retained; however, lumbar BMD decreased 3.8%. The hiker eventually regained this loss of lumbar BMD. A decrease in lumbar BMD despite engaging in load‐bearing activity was also observed in two recent case study reports of male thru‐hikers, suggesting a maladaptive response that warrants further investigation.

## 1 INTRODUCTION

Thru‐hiking can be defined as hiking the entirety of a long‐distance trail in one continuous journey or hiking season. This activity poses many logistical, mental, and physical challenges. Among the physical challenges are hiking many miles with a loaded backpack, managing overuse injuries and foot care, adapting to environmental stressors, and maintaining a sufficient caloric intake to fuel the effort.

Popular thru‐hikes throughout the world include the Camino de Santiago in Spain, Te Araroa in New Zealand, and the Appalachian Trail in the eastern United States. The Pacific Crest Trail (PCT) in the western United States is also an iconic thru‐hike. The PCT traverses 4265 km (2650 miles) approximately 200 km inland along the entire west coast of the United States from the Mexican border to the Canadian border. The total elevation gain/loss is estimated to be 149,175 m (489,418 ft.), with a low point of 43 m (141 ft.) and a high point of 4009 m (13,153 ft.). Despite the challenges, thru‐hike permits (required for trail sections >805 km) have averaged 7224 per year since 2021 (Pacific Crest Trail Association, 2026).

A number of case studies that include physiological data on PCT thru‐hikers have been published in recent years. These are summarized in Table 1. None of these previous case reports include a premenopausal female adult, and some of the individuals did not complete the entire route. The purpose of this case study was to quantitatively document changes in body composition and bone health of a young female adult who completed the PCT thru‐hike.

**TABLE 1:** Summary of previously published case studies of PCT thru‐hikers.

Ref #	Participant		PCT data		Body composition	Bone data	Main findings/other information
	Sex	Age	Distance	Days	Pre‐ to post‐changes	Pre‐ to post‐changes	
Present study	Female	24	4265 km	182	Mass: 71 to 61 kg %BF: 33.2% to 24.9% 52% drop in VAT	Total BMD: +1.8% Lumbar BMD: −3.8%	Dramatic losses in fat mass and visceral fat; lumbar BMD decreased while total BMD was retained; menstrual cycle dysfunction; vegetarian
Fasczewski et al. (2020)	Male	26	4265 km	140	Stable BMI (24.2 to 23.8 kg/m^2^) Stable %BF (11.8% to 8.9%)	Total BMD: −1.7%	Minimal cardiopulmonary changes; high levels of autonomous motivation and resilience
Heinbockel and Craighead (2021)	Male	25	4052 km	112	Stable BMI (23.7 to 23.4 kg/m^2^) Stable %BF (5.8% to 6.0%) Decrease in appendicular FM, but increase of trunk FM LM: −1.2%	Total BMD: no change Lumbar BMD: −5.2% Pelvis BMD: −3.8%	Decrease in endothelial function; high‐calorie, low‐quality diet
Saenz et al. (2024)	Female	62	1506 km	70	BMI: 21.6 to 19.5 kg/m^2^ %BF: 26% to 19.6% 10% loss of mass	NR	Chronic caloric deficit (RED‐S); unable to finish due to fatigue; vegetarian
Weiss et al. (2023)	Male	55	3767 km	128	Mass: 86.5 to 78.8 kg; %BF: 22.7% to 13.0% FM: −46% LM: +7%	Lumbar BMD: −8.6% Hip BMD: −1.0%	Chronic caloric deficit (RED‐S); increases in total and LDL cholesterol and triglycerides; vegetarian

Abbreviations: %BF, body fat percentage; BMD, bone mineral density; BMI, body mass index; FM, fat mass; LM, lean mass; NR, not reported; VAT, visceral adipose tissue.

## 2 MATERIALS AND METHODS

### 2.1 Design

This is a retrospective longitudinal case study to examine the change in body composition and bone health of a young adult female following completion of a PCT thru‐hike (see Data S1 for the CARE checklist).

### 2.2 Participant and nutrition

The case study participant was a former collegiate distance runner and was a recreationally‐active female of 24 years at the start of the thru‐hike and 25 years at the completion. She was a vegetarian, but consumed canned tuna at times throughout the hike. Although a daily nutrition log was not kept, she had photos of food resupplies and provided a retrospective dietary summary. Breakfasts consisted of Pop Tarts, energy or protein bars, rice crispy treats, dried cheese crisps, and occasionally cereal. Lunches and dinners consisted of mini bagels, avocado, vegan cream cheese, cheddar block cheese, tuna, tortillas, Nutella, chips, mashed potatoes, couscous, pasta, and occasionally ramen. Snacks included electrolyte drink mixes, gummy candy or fruit snacks, cookies, peanut butter, trail mix, and natural berries. When the trail passed through a town, meals would be in restaurants and food items were typically veggie omelets, hashbrowns, Beyond burgers, fresh vegetables, pizza, and ice cream. No dietary supplements were taken, but the hiker would occasionally take ibuprofen or acetaminophen as needed.

Pre‐ and post‐lab measurements were made at the request of the hiker for her own personal interest. Retrospectively, the hiker was contacted about using her data in a case study report. She consented verbally and in writing, and the Utah State University IRB approved the data dissemination (protocol #15247).

### 2.3 PCT thru‐hike

The participant began the hike on March 28, 2023 and completed it on September 25, 2023. The hike commenced from the southern terminus at Campo, CA and progressed to Kennedy Meadows, CA. There was a break in hiking from May 18 to May 30 for travel; hiking resumed at Chester, CA, and continued until reaching the Canadian border on August 16. The hiker then traveled by vehicle from the Canadian border back to Chester, CA, and resumed hiking south on August 21, reaching Kennedy Meadows, CA, on September 25. Consequently, the entire 4265 km (2650 mile) PCT was completed without missing any sections. Daily hiking distance varied, but was generally between 35 and 45 km and on occasion reached 60 km. Pack weight varied depending on climate and logistics, but was generally about 12–15 kg.

### 2.4 DXA measurement

Laboratory measurements occurred approximately 1 week before the hike began and 1 week after completion. Height was measured to the nearest 0.1 cm with a wall‐mounted stadiometer (Seca 222, Seca Corp., Ontario, CA), and body mass was measured to the nearest 0.1 kg with a digital scale (Seca 869, Seca Corp., Ontario, CA). The participant was shoeless and wearing shorts and a t‐shirt for these measurements. The participant rested in a supine position for a 6‐min whole‐body dual‐energy x‐ray absorptiometry (DXA) scan (Horizon‐W, Hologic, Inc., Marlborough, MA; APEX system software version 5.6.0.5) for body composition. This software defines the region of interest for visceral adipose tissue (VAT) as vertically between the pelvis and last rib and horizontally the inner edge of the abdominal muscle wall. The least significant change (LSC) 95% coefficient of variation (CV) for fat mass from this lab is 2.72%. Additional scans were done for bone mineral content (BMC) and bone mineral density (BMD) of the right and left femoral neck and the lumbar (L1‐L4) spine. The LSC 95% CV for BMD at the hip and lumbar spine from this lab is 2.13% and 1.78%, respectively. The DXA was calibrated prior to the measurement sessions using the manufacturer‐provided spine phantom. Body positioning was consistent with the manufacturer's instructions, and the same licensed bone densitometry operator performed both pre‐ and post‐assessments.

## 3 RESULTS

Retrospectively, the hiker reported having regular 26‐days menstrual cycles for the 2 years leading up to this thru‐hike; however, the cycle was lost within the first month of being on the PCT. Menstruation did not return until a couple months after completing the thru‐hike and was sporadic until March 2024, with return of regular cycles.

The hiker lost 10.0 kg of mass (from 71.0 to 61.0 kg), and body mass index dropped from 28.4 to 24.4 kg/m^2^ from the pre‐ to post‐measurement. Body fat percentage dropped from 33.2% to 24.9% with VAT cut in half. BMD was retained at the femoral neck (*z*‐score increase of 0.1), but decreased at the lumbar spine (*z*‐score decrease of 0.3). Details of body composition and bone changes are in Table 2.

**TABLE 2:** Body composition and bone changes following a Pacific Crest Trail thru‐hike.

Variable	Pre‐hike	Post‐hike	Change (%)
Body fat percent (%)	33.2	24.9	−8.3 (−25.0%)
Fat mass (kg)	23.7	15.3	−8.4 (−35.4%)
VAT mass (g)	444	213	−231 (−52.0%)
VAT volume (cm^3^)	480	230	−250 (−52.1%)
VAT area (cm^2^)	92.1	44.2	−47.9 (−52.0%)
Lean mass (kg)	45.6	43.8	−1.8 (−3.9%)
aSMI (kg/m^2^)	8.06	7.67	−0.4 (−4.8%)
Total BMC (kg)	2.2	2.2	0 (0%)
Total BMD (g/cm^2^)	1.184	1.205	0.021 (1.8%)
Lumbar BMD (g/cm^2^)	0.988	0.950	−0.038 (−3.8%)
Left FN BMD (g/cm^2^)	0.814	0.824	0.010 (1.2%)
Right FN BMD (g/cm^2^)	0.863	0.878	0.015 (1.7%)

Abbreviations: aSMI, appendicular skeletal muscle index; BMC, bone mineral content; BMD, bone mineral density; FN, femoral neck; VAT, visceral adipose tissue.

## 4 DISCUSSION

The hiker experienced a large decrease in fat mass, particularly VAT, with a relatively small loss in lean mass. This is generally regarded as an ideal cardiometabolic health outcome (Shao et al., 2025). Losing this amount of fat with only a small loss of fat‐free mass is difficult to achieve. Reviews suggest that 20%–40% of typical weight loss is comprised of fat‐free mass (Heymsfield et al., 2014; McCarthy & Berg, 2021); however, thru‐hiking long distances is not a “typical” weight loss strategy, and the large volume of exercise may have attenuated the autophagy pathway linked to loss of skeletal muscle mass (Martin‐Rincon et al., 2019).

However, this dramatic improvement in body composition came with a transient risk to bone health. Total body BMC and BMD were retained, but there was a loss in BMD at the lumbar spine. This was surprising because load‐bearing exercise is known to be beneficial for BMD (Guadalupe‐Grau et al., 2009), and one would expect hiking long distances with a backpack would increase rather than decrease BMD. Further, the BMD loss was localized to the lumbar spine. Close inspection of the other PCT case studies revealed a similar pattern of total BMD being retained with regional BMD losses, with the losses at the spine exceeding those at the hip (Heinbockel & Craighead, 2021; Weiss et al., 2023). Possible explanations for this unusual site‐specific decrease of BMD despite months of backpacking include the theory that bone cells become desensitized to prolonged repetitive exercise (i.e., thru‐hiking) (Turner & Robling, 2003), and there is less cortical bone and loading on the lumbar spine compared to the femoral neck, making the lumbar spine more susceptible to BMD loss (Mountjoy et al., 2018).

Recently, we were able to reassess the case study participant. This follow‐up assessment occurred 32 months after the post‐PCT measurement. Lumbar BMD was 1.002 g/cm^2^, nearly identical to her pre‐PCT measurement of 0.988 g/cm^2^ and a substantial improvement over her post‐PCT measurement of 0.950 g/cm^2^. Consequently, at least for this hiker, it appears possible to regain BMD loss associated with an arduous thru‐hike.

Although we do not have an account of the hiker's energy balance, the interrelatedness of her dietary intake, menstrual dysfunction, and reduced lumbar BMD suggests that our case study participant likely experienced relative energy deficiency in sport (RED‐S) (de Souza et al., 2019; de Souza et al., 2026; Mountjoy et al., 2018). RED‐S is defined as impaired physiological functioning caused by insufficient energy intake (Mountjoy et al., 2018). With negative energy balance, there are bioenergetic alterations that conserve energy at the expense of growth and reproduction. Chronic caloric deficit leads to decreases in the pulsatility of gonadotropin releasing hormone and leutinizing hormone; eventually, decreased estrogen levels and ‘functional hypothalamic amenorrhea’ lead to loss of bone mass (de Souza et al., 2019; de Souza et al., 2026; Mountjoy et al., 2018).

This case study was limited by retrospective data collection; a study was not pre‐planned. Consequently, there was no metabolic testing to estimate energy needs or blood work to measure hormone levels or biomarkers of bone turnover. Nevertheless, the study provides a unique glimpse into the adaptive physiology of a young, premenopausal female following completion of an ultra‐endurance backpacking trek.

## 5 CONCLUSION

In summary, the thru‐hiker lost large amounts of fat mass and VAT. A relatively small amount of lean mass was lost. However, there was a 3.8% transient loss of lumbar BMD at an age in life when BMD should still be increasing, or at least not decreasing (Chew & Clarke, 2018). RED‐S was likely the key contributing factor. Two PCT case studies of men show even larger lumbar BMD losses of 5.2% (Heinbockel & Craighead, 2021) and 8.6% (Weiss et al., 2023). Our case study participant may have had more rest and travel days interspersed than the men, and this may have helped to attenuate the BMD loss. Regardless, although the sample of case studies is small, there appears to be a consistent and concerning pattern of reduced lumbar BMD associated with long thru‐hikes despite walking many hours with a load. This maladaptive physiological response to ultra‐distance load‐bearing activity warrants future investigation with a greater sample size and additional laboratory analyses.

## AUTHOR CONTRIBUTIONS

**Dale R. Wagner:** Conceptualization; data curation; formal analysis; investigation; methodology. **Edward M. Heath:** Conceptualization; methodology. **Macy Gustavus:** Data curation; investigation.

## FUNDING INFORMATION

This research was conducted without any financial support.

## CONFLICT OF INTEREST STATEMENT

The authors report no conflict of interest.

## ETHICS STATEMENT

The study was approved by Utah State University IRB (protocol #15247).

## Supporting information


Data S1:

## Data Availability

All relevant data are in the manuscript. Additional information is not available to protect the anonymity of the case study participant.
