# Supplementary material for: Case study: Body composition and bone changes following a thru‐hike of the Pacific Crest Trail by a young female adult
Source: Physiol Rep. 2026 Aug 9;14(15):e71058. doi: 10.14814/phy2.71058 (PMC13454730; doi:10.14814/phy2.71058)
Supplement: Supplementary file 1 [file PHY2-14-e71058-s001.pdf]

CARE
case report guidelines

CARE Checklist of information to include when writing a case report

(cc) BY-NC-ND

Topic	Item	Checklist item description	Reported on Line
Title	1	The diagnosis or intervention of primary focus followed by the words "case report"	1 (Title)
Key Words	2	2 to 5 key words that identify diagnoses or interventions in this case report, including "case report"	27-28 (keywords)
Abstract (no references)	3a	Introduction: What is unique about this case and what does it add to the scientific literature?	18-19
	3b	Main symptoms and/or important clinical findings	20-23
	3c	The main diagnoses, therapeutic interventions, and outcomes	23-24
	3d	Conclusion—What is the main "take-away" lesson(s) from this case?	24-26
Introduction	4	One or two paragraphs summarizing why this case is unique (**may include** references)	44-49
Patient Information	5a	De-identified patient specific information.	56-58
	5b	Primary concerns and symptoms of the patient.	104-108
	5c	Medical, family, and psycho-social history including relevant genetic information	104-105
	5d	Relevant past interventions with outcomes	N/A (no intervention)
Clinical Findings	6	Describe significant physical examination (PE) and important clinical findings.	85-102; table 2
Timeline	7	Historical and current information from this episode of care organized as a timeline	74-83
Diagnostic Assessment	8a	Diagnostic testing (such as PE, laboratory testing, imaging, surveys).	85-102
	8b	Diagnostic challenges (such as access to testing, financial, or cultural)	85-86
	8c	Diagnosis (including other diagnoses considered)	143-152
	8d	Prognosis (such as staging in oncology) where applicable	107-108; 137-142
Therapeutic Intervention	9a	Types of therapeutic intervention (such as pharmacologic, surgical, preventive, self-care)	N/A (no intervention)
	9b	Administration of therapeutic intervention (such as dosage, strength, duration)	N/A (no intervention)
	9c	Changes in therapeutic intervention (with rationale)	N/A (no intervention)
Follow-up and Outcomes	10a	Clinician and patient-assessed outcomes (if available)	137-142
	10b	Important follow-up diagnostic and other test results	137-142
	10c	Intervention adherence and tolerability (How was this assessed?)	N/A (no intervention)
	10d	Adverse and unanticipated events	N/A (no intervention)
Discussion	11a	A scientific discussion of the strengths AND limitations associated with this case report	153-157
	11b	Discussion of the relevant medical literature **with references**.	115-152
	11c	The scientific rationale for any conclusions (including assessment of possible causes)	159-170
	11d	The primary "take-away" lessons of this case report (without references) in a one paragraph conclusion	159-162
Patient Perspective	12	The patient should share their perspective in one to two paragraphs on the treatment(s) they received	N/A (no intervention)
Informed Consent	13	Did the patient give informed consent? Please provide if requested	Yes ☒ No ☐
